# Characterization of novel mouse models to study the role of necroptosis in aging and age-related diseases

**DOI:** 10.1007/s11357-023-00955-7

**Published:** 2023-10-04

**Authors:** Ramasamy Selvarani, Hoang Van Michelle Nguyen, Nidheesh Thadathil, Roman F. Wolf, Willard M. Freeman, Christopher D. Wiley, Sathyaseelan S. Deepa, Arlan Richardson

**Affiliations:** 1 Biochemistry & Molecular Biology, University of Oklahoma Health Sciences Center, Oklahoma City, OK USA; 2 Nutritional Sciences, University of Oklahoma Health Sciences Center, Oklahoma City, OK USA; 3 Okalahoma Veteran Affairs Medical Center, Oklahoma City, OK USA; 4 Okalahoma Medical Research Foundation, Oklahoma City, OK USA; 5 Jean Mayer USDA Human Nutrition Center On Aging, Boston, MA USA; 6 Stephenson Cancer Center, Oklahoma City, OK USA

**Keywords:** Necroptosis, Receptor-interacting protein kinase, Ripk3, Mixed lineage kinase domain like protein, Mlkl, Inflammation, Knockin mice, Chronic liver diseases, Fibrosis, Steatosis, Cell senescence

## Abstract

To study the impact of necroptosis-induced chronic inflammation on age-related diseases and aging, two knockin mouse models (*Ripk3*-KI and *Mlkl*-KI) were generated that overexpress two genes involved in necroptosis (*Ripk3* or *Mlkl*) when crossed to Cre transgenic mice. Crossing *Ripk3*-KI or *Mlkl*-KI mice to albumin-Cre transgenic mice produced hepatocyte specific *hRipk3*-KI or *hMlkl*-KI mice, which express the two transgenes only in the liver. Ripk3 and Mlkl proteins were overexpressed 10- and fourfold, respectively, in the livers of the *hRipk3*-KI or *hMlkl*-KI mice. Treating young (2-month) *hRipk3*-KI or *hMlkl*-KI mice with carbon tetrachloride (CCl_4_), a chemical inducer of oxidative stress, resulted in increased necroptosis (Mlkl-oligomers) and inflammation in the liver compared to control mice receiving CCl_4_. Mlkl-oligomerization also was significantly increased in old (18-month) *hRipk3*-KI and *hMlkl*-KI mice compared to old control (Cre negative, *Ripk3*-KI and *Mlkl*-KI) mice. The increase in necroptosis was associated with an increase in inflammation, e.g., inflammatory cytokines (TNFα, IL-6) and macrophage markers (F4/80, CD68). Importantly, steatosis (triglycerides) and fibrosis (e.g., picrosirius red staining, hydroxyproline levels, and transcripts for TGFβ, Col1α1, and Col3α1) that increase with age were significantly higher in the livers of the old *hRipk3*-KI or *hMlkl*-KI mice compared to old control mice. In addition, markers of cellular senescence were significantly increased in the livers of the old *hRipk3*-KI and *hMlkl*-KI mice. Thus, the first mouse models have been developed that allow researchers to study the impact of inducing necroptosis in specific cells/tissues on chronic inflammation in aging and age-related diseases.

**Supplementary Information:**

The online version contains supplementary material available at 10.1007/s11357-023-00955-7.

## Introduction

Chronic, low-grade inflammation (inflammaging) is a prominent characteristic of aging and poses a significant risk to the health and longevity of elderly individuals [1]. Furthermore, inflammation is a major contributing factor to various age-related diseases, including type 2 diabetes, cardiovascular disease, cancer, neurodegenerative diseases, and frailty, among others [2]. Despite the importance of inflammation in aging and age-associated diseases, the molecular mechanism(s)/pathway(s) responsible for chronic, low-grade inflammation seen in aging is poorly understood. 

One potential factor causing inflammaging are damage-associated molecular patterns (DAMPs), which have been shown to play a major role in inflammation [3]. Circulating mitochondrial DNA, which is a DAMP, increases with age in humans and is correlated with increased inflammation [4]. Necroptosis is a pathway of programmed/regulated necrosis, which generates DAMPs and has been shown to play an important pro-inflammatory role [5]. Necroptosis is induced by various stimuli (e.g., TNFα, oxidative stress, mTOR activation), which activate the receptor-interacting protein kinase 1 (Ripk1), Ripk3, and mixed lineage kinase domain like (Mlkl) proteins through phosphorylation. Phosphorylated Mlkl oligomerizes and binds to and disrupts the plasma membrane of cells, releasing DAMPs. The DAMPs bind to cell surface receptors on innate immune cells triggering the production of proinflammatory cytokines such as TNF-α, IL-6, and IL-1β leading to inflammation [3].

Necroptosis has been identified as a significant contributor to chronic inflammation in several studies [6–10]. Targeting necroptosis through Ripk1, Ripk3, or Mlkl either genetically (knockout mice) or pharmacologically can reduce inflammation in various mouse models [11]. For example, knocking out Ripk3 reduced inflammation in a mouse model of atherosclerosis [6], in intestinal epithelial cells of FADD [10], in caspase8 deficient mice [10], and in methionine-choline deficient diet-induced liver steatosis [12]. The Ripk1 inhibitor necrostatin-1 (Nec-1) effectively blocks necroptosis and inflammation in a mouse model of dextran sulfate sodium-induced colitis [13], protects the brain against ischemic necroptosis [14], reduces oligodendrocyte cell death in an in vivo model of multiple sclerosis [15], and reduces neuronal loss in transgenic Alzheimer’s mouse models [16]. In addition, knocking out Mlkl has been shown to reduce neuroinflammation in the Japanese encephalitis virus mouse model [17]. Our group recently showed that knocking out either Ripk3 or Mlkl attenuated the increase in inflammation and severity of hepatocellular carcinoma in the livers of mice fed a choline-deficient, high-fat diet [18].

Our group was the first to show that necroptosis increased with age in white adipose tissue, which was associated with increased markers of inflammation [19]. We found that dietary restriction attenuated both necroptosis and inflammation. Subsequently, we showed that necroptosis increased with age in the liver [20] and the brain [21] and was increased in the liver of *Sod1*^*−/−*^ mice, a mouse model of accelerated aging [22]. We found that treating mice with Nec-1 s reduced necroptosis in the liver [20] and the brain [21] of old mice and the liver of *Sod1*^*−/−*^ mice [22] and, importantly, reduced markers of inflammation in these tissues of the mice. Based on these data, it appears that necroptosis plays a role in inflammaging.

Although a large number of studies have shown that blocking/reducing necroptosis reduces inflammation in various disease conditions, there are only a few reports that have examined the effect of inducing necroptosis. All such studies have been conducted in cell cultures, e.g., overexpressing Ripk3 in C2C12 myoblasts cells [23] and cardiomyocytes [24] or Mlkl in mouse embryonic fibroblasts [25] and hepatocytes [26]. However, there are no mouse models that allow investigators to study in vivo the impact of Ripk3 and Mlkl grain of function on necroptosis-induced inflammation. Therefore, we developed two novel knockin (KI) mouse models that can be used to overexpress either Ripk3 or Mlkl in specific cells/tissues. By crossing these KI mouse models to albumin-Cre transgenic male mice, we show that the expression of Ripk3 or Mlkl is increased 10- or fourfold, respectively, only in the liver. When these mice are exposed to mild stress of carbon tetrachloride (CCl_4_) or aging, they show an increase in necroptosis as well as markers of inflammation in the liver.

## Methods

### Animals

All procedures were approved by the Institutional Animal Care and Use committee at the Oklahoma City Veterans Affairs Health Care System Animal Facility. The knockin (KI) mice were generated in C57BL/6 J mice by ViewSolid Biotech Inc. (Oklahoma City, OK) using the transgenic constructs shown in Supplementary Figure S1A. The transgene contained the cDNA to either *Ripk3* or *Mlkl*, which was tagged with a flag-tag (3 ×) sequence on the 3′-end of the cDNA and a stop cassette (3 ×) flanked by loxP sites inserted at the 5′ end of the transgene. The sequences of the two transgenes are shown in Supplementary Table S1. The gRNA is designed to guide the CRISPR/Cas9-mediated homology-directed repair to the intron region between exon1 and exon2 of mouse *Rosa26* gene. Germline KI mice were generated by crossing male hemizygous mice (either *Ripk3*-KI or *Mlkl*-KI) produced to female C57BL/6 J mice obtained from Jackson Laboratories (Bar Harbor, Main). The *Ripk3*-KI and *Mlkl*-KI mice were identified by PCR as shown in Supplementary Figure S1B,C using DNA from ear notches for PCR analysis with the primers shown in Supplementary Table S2 for the left homologous region (LHR 528 bp) and the right homologous region (RHR 670 bp). The *Ripk3*-KI mice generated litters of 4 to 6 pups, and the *Mlkl*-KI mice generated litters of 4 to 10 pups. The hemizygous *Ripk3*-KI or *Mlkl*-KI female mice were crossed to male mice homozygous for albumin-Cre transgenic obtained from Jackson laboratory (Bar Harbor, Main, stock no#003574) to produce mice that express *Ripk3* or *Mlkl* specifically in hepatocytes, which are designated as *hRipk3*-KI or *hMlkl*-KI mice, respectively. The Cre under the regulation of the albumin promoter is expressed in hepatocytes of embryos starting from E14 to 21 days onwards [27]. Approximately 50% of pups produced by this cross were either *hRipk3*-KI or *hMlkl*-KI mice as expected based on Mendelian inheritance. Supplementary Figure S1D, E shows there was no difference in the body weights of the *hRipk3*-KI or *hMlkl*-KI mice from 2 to 14 months of age compared to their controls, either *Ripk3*-KI or *Mlkl*-KI mice, which had not been crossed to the albumin-Cre transgenic mice.

The mice were generated and maintained in the animal facility at the Oklahoma City Veterans Affairs Health Care System Animal Facility. Two- and 18-month-old male mice were used in these experiments. The mice were group housed in ventilated cages at 20 ± 2 °C, on a 12-h/12-h dark/light cycle, and fed a laboratory rodent chow (5053 Pico Lab, Purina Mills, Richmond, Indianapolis) ad libitum*.* After the mice were euthanized, blood and liver tissue were collected, samples frozen in liquid nitrogen, and stored at − 80 °C until analyzed.

### Administration of CCl_4_

Two-month-old mice were given an acute dose of CCl_4_ as previously described [28]. CCl_4_ (Sigma, St. Louis, MO) was dissolved in olive oil (50:50) and given as a single intraperitoneal injection of CCl_4_/olive oil (50 µL/mouse) resulting in a dose of 2 mL CCl_4_/kg body weight. Mice were euthanized 24 h after receiving the CCl_4_/olive oil or olive oil control.

### RNA isolation and quantification of mRNA transcripts

Total RNA was extracted using the RNeasy kit (Qiagen, Valencia, CA) from 20 mg of frozen liver tissue as described previously [20]. RT-PCR was performed using a high-capacity cDNA reverse transcription kit (Thermo Fisher Scientific, Waltham, MA), and quantitative real-time PCR was performed with ABI Prism using Power SYBR Green PCR Master Mix (Thermo Fisher Scientific, Waltham, MA). The primers used for RT-PCR analysis are given in Supplementary Table S3 in the supplement. The transcript levels of genes involved in various processes that were measured in this study are as follows: macrophage markers (F4/80, CD68, CD206), inflammatory cytokines (TNFα, IL-1β, IL-6), fibrosis markers (TGFβ, Col1α1, and Col3α1), cell senescence markers (p16, p21), and senescence-associated secretory phenotype (SASP) factors (PAI-1, CXCl-1, CXCl-8, CXCl-10, MMP-9, MMP-12, UPAR, p19, GDF-15, p53). The relative mRNA levels were determined by a series of calculations. First, the delta CT(∆CT) of the target gene is calculated by subtracting the ∆CT value of the reference gene (β-microglobulin). Next, the delta delta CT (∆∆CT) is obtained by subtracting the ∆CT value of the target sample from the average of ∆CT value of the control samples. Finally, to calculate the fold changes in mRNA levels, we use the formula involving the exponentiation of 2 to the power of negative ∆∆CT (2^−ΔΔ*Ct*^). The fold change is determined by comparing the average ∆CT of the experimental group to the average of ∆CT of the control group.

### Western blotting

Western blotting was performed as described previously [20]. Fifty milligrams of tissue was homogenized in RIPA lysis buffer (Thermo Fisher Scientific, Waltham, MA) containing 2 mM phenylmethylsulfonyl fluoride and protease inhibitor cocktail (GoldBio, St. Louis, MO). Protein concentration was determined with Bio-Rad BCA Protein Assay (Hercules, CA). Western blotting was performed using 20 mg protein on an SDS-PAGE gel and transferred onto nitrocellulose membrane. Images were taken using a Chemidoc imager (Bio-Rad, Hercules, CA) and quantified using ImageJ software (U.S. National Institutes of Health, Bethesda, MD). The following primary antibodies were used: Ripk1 and Ripk3 from Novus biologicals (Centennial, CO), Mlkl from Millipore Sigma (St. Louis, MO) and GAPDH, β-tubulin, and β-actin antibody from Sigma-Aldrich (St. Louis, MO). HRP-linked anti-rabbit IgG from Cell Signaling Technology (Danvers, MI) was used as a secondary antibody. To quantify the western blots, the intensity of protein of interest was divided by the corresponding control band intensity (e.g., either β-actin, β-tubulin, or GAPDH). Subsequently, the intensity of the band on each sample was then divided by the average intensity of control group, thereby expressing the data as a fold change in the proteins of interest.

MLKL-oligomers in the liver were detected using western blots under non-reducing conditions as we have previously described [22, 29]. Briefly, the liver tissue was homogenized in HEPES buffer (pH 7.4), and protein in the homogenate was quantified using Bradford Bio-Rad BCA Protein Assay (Hercules, CA). Protein samples were prepared using 2 × Laemmli buffer without any reducing agents to maintain the proteins under non-reducing conditions. Forty micrograms of protein was used, and gels were run under non-reducing conditions without SDS in running buffer and on 7.5% poly-acrylamide gel. MLKL-oligomers were detected and quantified on the gels using the antibody to MLKL as described above for oligomers larger than 200 kDa.

### Hydroxyproline assay

The hydroxyproline content of the liver was measured as described by [29]. Liver tissue (~ 250 mg) was pulverized using liquid nitrogen and digested in 6-M hydrochloric acid overnight at 110 °C. Ten milliliters of the digest was mixed with 150 µL of isopropanol, 75 µL of solution A (1:4 mix of 7% Chloramine T (Sigma-Aldrich, St. Louis, MO), and acetate citrate buffer (containing 57 g sodium acetate anhydrous, 33.4 g citric acid monohydrate, 435 mL 1 M sodium hydroxide, and 385 mL isopropanol in 1 L of buffer). The mixture was vigorously mixed and incubated at room temperature for 10 min. Solution B (3:13 mix of Ehrlich’s reagent and isopropanol) was added, and the solution incubated at 58 °C for 30 min. The reaction was stopped by placing on ice for 10 min, and the absorbance at 558 nm was measured in a Spectra Max M2 spectrophotometer (Molecular Devices, San Jose, CA). The absorbance values were converted into µg units by standard curve using the standards and expressed as micrograms of hydroxyproline per gram of tissue.

### Picrosirius red staining

Formalin-fixed liver tissue was embedded in paraffin, and 4-µm sections were generated using a microtome. Picrosirius red staining was conducted using standard protocol at the Imaging Core facility at the Oklahoma Medical Research Foundation. Briefly, formalin-fixed sections were deparaffinized and stained with Picrosirius Red for 1 h. Excess picrosirius red was removed by rinsing in acidified water, and sections were dehydrated with ethanol and cleared with xylene. The images were taken using a Nikon TI Eclipse microscope (Nikon, Melville, NY) for 3 random fields per sample and quantified using ImageJ software.

### Hemoxylin and eosin staining for lipid droplets

Formalin-fixed liver tissue was embedded in paraffin, and 4-µm sections were generated using a microtome. Hematoxylin and eosin (H&E) staining was performed on the tissue samples using the standard procedure at the Stephenson Cancer Centre Tissue Pathology Core. H&E-stained sections were digitally scanned at 10 × and 20 × magnifications using Nikon Ti Eclipse microscope (Nikon, Melville, NY).

### Plasma alanine transaminase assay (ALT) measurement

Whole blood was collected in EDTA coated tubes and left undisturbed on ice for 15–30 min. Plasma was obtained by centrifuging at 1000–2000 × g for 20 min at 4 °C and collecting the supernatant. Plasma levels of ALT were measured using alanine transaminase colorimetric activity assay kit from Cayman Chemical Company (Ann Arbor, MI) following the manufacturer’s instructions.

### Liver triglyceride measurement

The triglyceride content of liver was measured using triglyceride colorimetric activity assay kit from Cayman Chemical Company (Ann Arbor, MI) following manufacturer’s instructions. The lipid content of the liver was expressed as milligrams per gram of liver tissue.

## Results

### Characterization of the knockin (KI) mouse models

**Fig. 1:**
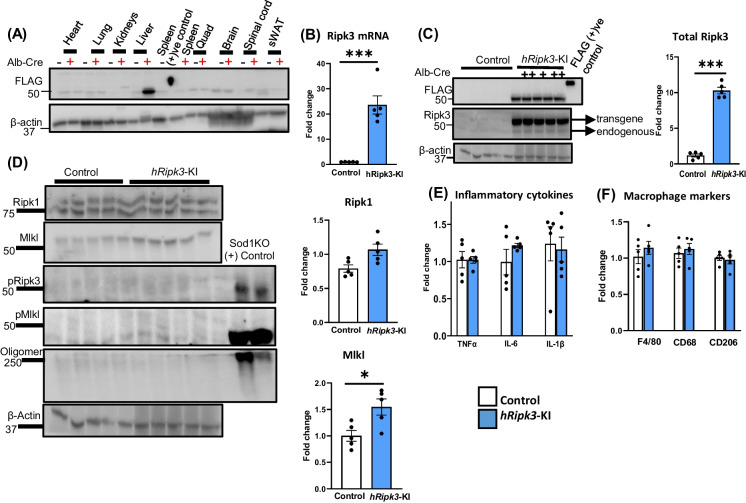
Characterization of *hRipk3*-KI mouse model. The expression of Ripk3, markers of necroptosis, and inflammation were measured in 2-month-old control (*Ripk3*-KI mice, white bars) or *hRipk3*-KI mice (blue bars). Panel **A**: Expression of the *Ripk3*-transgene in various tissues as measured by western blotting using an antibody to flag (minus sign and plus sign represent *Ripk3*-KI mice or *hRipk3*-KI mice, respectively). Panel **B**: Transcript levels of Ripk3 normalized to β-microglobulin expressed as fold change. Panel **C**: Western blots showing the expression of the Ripk3 transgene and endogenous Ripk3 using an antibody to flag-tag or Ripk3, respectively. The graph to the right shows the quantification of total Ripk3 (normalized to β-actin) from the western blot expressed as fold change. Panel **D**: Western blots for Ripk1, Mlkl, pRipk3, pMlkl, Mlkl-oligomers, and β-actin using antibodies described in the “Methods” section. Tissue homogenates from *Sod1*^*−/−*^ (Sod1KO) were used as a control for measuring pRipk3, pMlkl, and Mlkl-oligomers. The graphs to the right show the fold change in Ripk1 and Mlkl normalized to β-actin from the western blots. Panel **E**: Transcript levels of TNFα, IL-1β, and IL-6 normalized to β-microglobulin expressed as fold change. Panel **F**: Markers of total macrophages (F4/80), proinflammatory M1 macrophages (CD68), and anti-inflammatory M2 macrophages (CD206) normalized to β-microglobulin expressed as fold change. Data were obtained from 5 mice per group, expressed as the mean ± SEM, and statistically analyzed using a Student *t*-test. ***p ≤ 0.0005

The transgene constructs illustrated in Supplementary Figure S1A were inserted into mouse *Rosa26* locus with the Ripk3- or Mlkl-transgene being expressed under the control of the endogenous Rosa26 promoter when the stop cassette is removed after crossing to a Cre-transgenic mouse. In this study, the *Ripk3*-KI and *Mlkl*-KI mice were crossed to albumin-Cre transgenic mice to express either Ripk3 or Mlkl specifically in the liver, which we designate as *hRipk3*-KI or *hMlkl*-KI mice, respectively. The expression of the transgenes were measured by the flag-tag. As shown in Supplementary Figure S1D,E, we observed no difference in the body weights of the *hRipk3*-KI or *hMlkl*-KI mice and their KI, controls (either *Ripk3*-KI or *Mlkl*-KI mice) from 2 to 14 months of age. We first measured expression of the transgenes in various tissues of 2-month-old mice. As shown in Fig. 1A, the Ripk3-transgene was expressed only in the liver tissue of *hRipk3*-KI mice and was not expressed in any tissues of the control, *Ripk3*-KI mice. Figure 1B shows that the level of Ripk3 mRNA (both the transgene and endogenous gene) was dramatically increased (~ 25-fold) in the livers of *hRipk3*-KI mice compared to control, *Ripk3*-KI mice. Next, we measured the level of Ripk3 protein in the livers of the *hRipk3*-KI and control mice. As can be seen from Fig. 1C (middle panel), two bands cross-react with the antibody to Ripk3 in the *hRipk3*-KI mice: a lower band consisting of the endogenous Ripk3 and an upper band for the Ripk3-transgene, which contains the flag-tag. The graph quantifying the western blot data shows that total Ripk3 protein levels in the livers of the h*Ripk3*-KI mice were ~ tenfold higher than that found in control mice. As shown in Fig. 1D (and graphs), we observed no significant change in Ripk1 levels; however, we observed a small, but significant increase in Mlkl levels in the livers of the *hRipk3*-KI mice. To determine if the overexpression of Ripk3 leads to necroptosis, we measured the levels of phospho-Ripk3 (pRipk3), phospho-Mlkl (pMlkl), and Mlkl-oligomers in the *hRipk3*-KI and control mice (Fig. 1D). We were surprised to find that none of the markers of necroptosis was induced in the *hRipk3*-KI mice even though Ripk3 levels were ~ tenfold higher in the *hRipk3*-KI mice. To confirm that necroptosis was not induced in the *hRipk3*-KI mice, we measured various markers of inflammation and macrophage activation, which would arise from DAMPs released from necroptotic cells. Figure 1E shows the expression of transcripts of TNFα, IL-6, and IL-1β, which are induced by necroptosis [29] and were not significantly different in the livers of *hRipk3*-KI and control mice. Similarly, we found no significant difference in markers for total macrophages (F4/80), proinflammatory M1 macrophages (CD68), and anti-inflammatory M2 macrophages (CD206) in the liver tissue from *hRipk3*-KI compared to control mice. Histopathological analysis of the liver tissue from *hRipk3*-KI mice was normal (Supplementary Figure S2A).

**Fig. 2:**
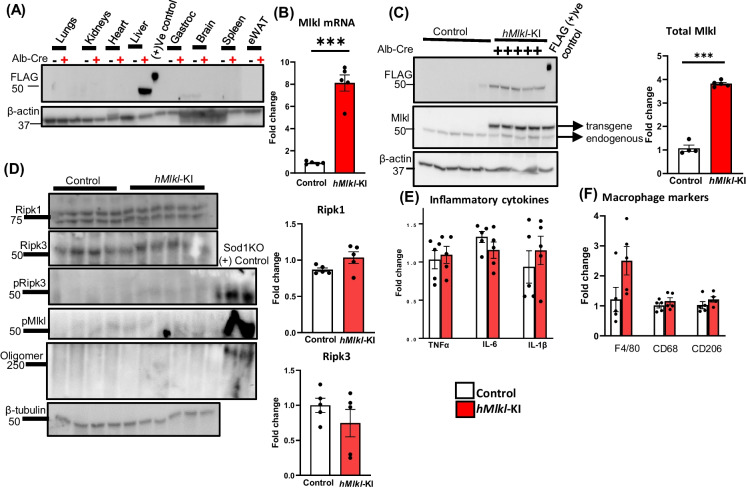
Characterization of the *hMlkl*-KI mouse model. The expression of Mlkl, markers of necroptosis, and inflammation were measured in 2-month-old control (*Mlkl*-KI mice, white bars) or *hMlkl*-KI mice (red bars). Panel **A**: Expression of the *Mlkl*-transgene in various tissues as measured by western blots using an antibody to flag (minus sign and plus sign represent *Mlkl*-KI mice or *hMlkl*-KI mice, respectively). Panel **B**: Transcript levels of Mlkl normalized to β-microglobulin expressed as fold change. Panel **C**: Western blots showing the expression of the Mlkl transgene and endogenous Mlkl using an antibody to flag-tag or Mlkl, respectively. The graph to the right shows the quantification of total Mlkl (normalized to β-actin) from the western blot expressed as fold change. Panel **D**: Western blots for Ripk1, Ripk3, pRipk3, pMlkl, Mlkl-oligomers, and β-tubulin using antibodies described in the “Methods” section. Tissue homogenates from *Sod1*^*−/−*^ (Sod1KO) were used as a control for measuring markers of necroptosis (pRipk3, pMlkl, and Mlkl-oligomers). The graphs to the right show the quantification of Ripk1 and Ripk3 normalized to β-tubulin from the western blots expressed as fold change. Panel **E**: Transcript levels of TNFα, IL-6, and IL-1β normalized to β-microglobulin expressed as fold change. Panel **F**: Markers of total macrophages (F4/80), proinflammatory M1 macrophages (CD68), and anti-inflammatory M2 macrophages (CD206) normalized to β-microglobulin expressed as fold change. Data were obtained from 5 mice per group, expressed as the mean ± SEM, and statistically analyzed using a Student *t*-test. ***p ≤ 0.0005

The characterization of *hMlkl*-KI mice is shown in Fig. 2. No tissues of control, *Mlkl*-KI mice, show any detectable expression of the Mlkl-transgene (flag-tag), and the expression of the Mlkl-transgene was only detectable in the liver of the *hMlkl*-KI mice. The levels of Mlkl mRNA were increased ~ eightfold in the livers of *hMlkl*-KI mice compared to control, *Mlkl*-KI mice (Fig. 2B), and the total Mlkl protein levels were increased ~ fourfold (Fig. 2C). As shown in Fig. 2D, we observed no significant change in protein levels of Ripk1 or Ripk3 levels in the *hMlkl*-KI mice. The *hMlkl*-KI mice also showed no detectable levels of markers of necroptosis (pRipk3, pMlkl, Mlkl-oligomers) in the liver. These data combined with no significant change in the expression of proinflammatory cytokines (Fig. 2E) or macrophages (Fig. 2F) in the liver indicate that the ~ fourfold overexpression of Mlkl in the *hMlkl*-KI mice did not lead to increased necroptosis. Histopathological analysis of liver tissue from *hMlkl*-KI mice was normal (Supplementary Figure S2B).

In summary, our data show that the *hRipk3*-KI or *hMlkl*-KI mice generated overexpress either the Ripk3 or Mlkl transgene specifically in the liver and not in any other tissue. To our surprise, we found that overexpressing these two genes, which are involved in necroptosis, did not induce necroptosis in the livers of the 2-month-old mice. Because necroptosis is triggered by the induction Ripk1-phosporylation, we conclude that factors triggering necroptosis were not present or minimal in non-stressed, young mice. Therefore, the following experiments were designed to study the impact of a mild oxidative stress on the induction of necroptosis in the livers of the *hRipk3*-KI and *hMlkl*-KI mice because oxidative stress has been shown to induce necroptosis [22].

### Carbon tetrachloride (CCl_4_) treatment induces markers of necroptosis in the livers of *hRipk3*-KI and *hMlkl*-KI mice

**Fig. 3:**
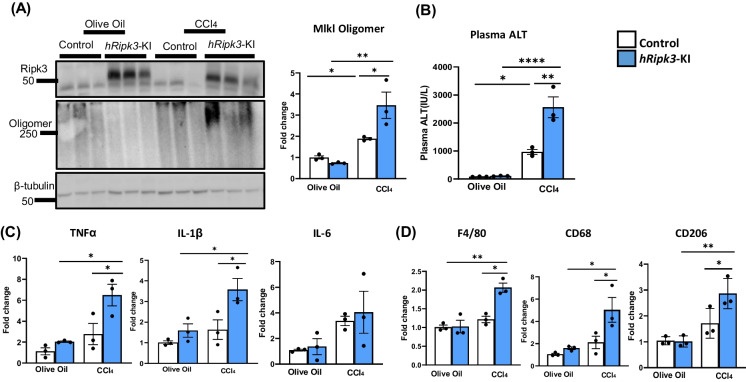
Effect of CCl_4_ on necroptosis and inflammation in the livers of *hRipk3*-KI mice. Control (*Ripk3*-KI mice, white bars) and hepatic *hRipk3*-KI (blue bars) mice were treated with olive oil or olive oil containing CCl_4_ as described in the “Methods” section. Panel **A**: Western blots of MLKL-oligomers and the graph to the right shows the quantification of MLKL-oligomers normalized to β-tubulin from the western blots expressed as fold change. Panel **B**: Plasma ALT levels (IU/L). Panel **C**: Transcript levels of TNFα, IL-1β, and IL-6 normalized to β-microglobulin expressed as fold change. Panel **D**: Markers of total macrophages (F4/80), proinflammatory M1 macrophages (CD68), and anti-inflammatory M2 macrophages (CD206) normalized to β-microglobulin expressed as fold change. Data were obtained from 3–6 mice per group, expressed as the mean ± SEM, and statistically analyzed using ANOVA. ****p ≤ 0.0001, **p ≤ 0.005, *p ≤ 0.05

CCl4 is known to induce oxidative stress resulting in hepatotoxic damage [30]. CCl_4_ was administered to male *Ripk3*-KI and *hRipk3*-KI mice at 2 months of age using the protocol described by Huang et al. [28], which used a mild acute dose to induce liver damage in vivo. The data in Fig. 3A show a significant induction of Mlkl-oligomers in the livers of CCl_4_-treated control, *Ripk3*-KI mice. Importantly, we observed a further increase (~ twofold) in Mlkl-oligomerization in the livers of CCl_4_-treated *hRipk3*-KI mice. Overexpressing Ripk3 in the liver resulted in a significant increased increase in TNFα and IL-1β mRNA levels when *hRipk3*-KI mice were treated with CCl_4_ compared to control mice (Fig. 3C). CCl_4_ treatment induced a similar increase in IL-6 mRNA levels in *hRipk3*-KI and control mice. CCl_4_ treatment also induced a significant increase in total macrophages (F4/80), proinflammatory M1 macrophages (CD68), and anti-inflammatory M2 macrophages (CD206) in *hRipk3*-KI mice compared to control mice (Fig. 3D). Thus, in response to CCl_4_ stress, the livers of the 2-month-old *hRipk3*-KI mice exhibit increased necroptosis and inflammation. We also measured the effect of overexpressing Ripk3 on plasma ALT levels, which is a marker of liver damage. As shown in Fig. 3B, the CCl_4_-treated *hRipk3*-KI mice showed over a twofold increase in ALT levels compared to control mice treated with CCl_4_.

**Fig. 4:**
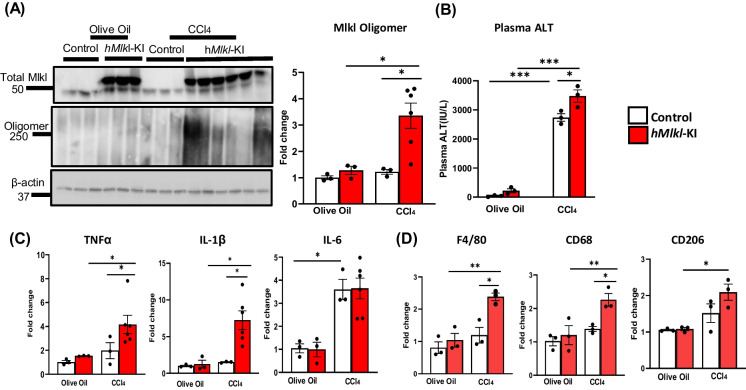
Effect of CCl_4_ induced oxidative stress on necroptosis and inflammation in the livers of *hMlkl*-KI mice. Control (*Mlkl*-KI mice, white bars) and *hMlkl*-KI mice (red bars) were treated with olive oil or olive oil containing CCl_4_ as described in the “Methods” section. Panel **A**: Western blots of MLKL-oligomers and the graph to the right shows the quantification of MLK-oligomers normalized to β-tubulin from the western blots expressed as fold change. Panel **B**: Plasma ALT levels (IU/L). Panel** C**: Transcript levels of TNFα, IL-1β, and IL-6 normalized to β-microglobulin expressed as fold change. Panel** D**: Markers of total macrophages (F4/80), proinflammatory M1 macrophages (CD68), and anti-inflammatory M2 macrophages (CD206) normalized to β-microglobulin expressed as fold change. Data were obtained from 3 to 6 mice per group, expressed as the mean ± SEM, and statistically analyzed using ANOVA. ***p ≤ 0.0005, **p ≤ 0.005, *p ≤ 0.05

Figure 4 shows the effect of CCl_4_ on 2-month-old *hMlkl-*KI mice. In these mice, CCl_4_ treatment showed only a small but not significant induction of Mlkl-oligomers in the control, *Mlkl*-KI mice (Fig. 4A). However, CCl_4_ treatment induced a dramatic increase (~ threefold) in Mlkl-oligomers. CCl_4_ treatment of the *hMlkl-*KI mice was also associated with an increase in the induction of TNFα and IL-1β mRNA levels (Fig. 4C), increased markers of total macrophages (F4/80) and proinflammatory M1 macrophages (CD68) (Fig. 4D), and a slight, but significant, increased liver damage as measured by plasma ALT levels (Fig. 4B). Thus, as with the *hRipk3-*KI mice, 2-month-old *hMlkl-*KI mice show increased induction of necroptosis when treated with CCl_4_ that leads to increased inflammation and liver damage.

### Necroptosis, inflammation, steatosis, and fibrosis are increased in the livers of old male *hRipk3*-KI and *hMlkl*-KI mice

**Fig. 5:**
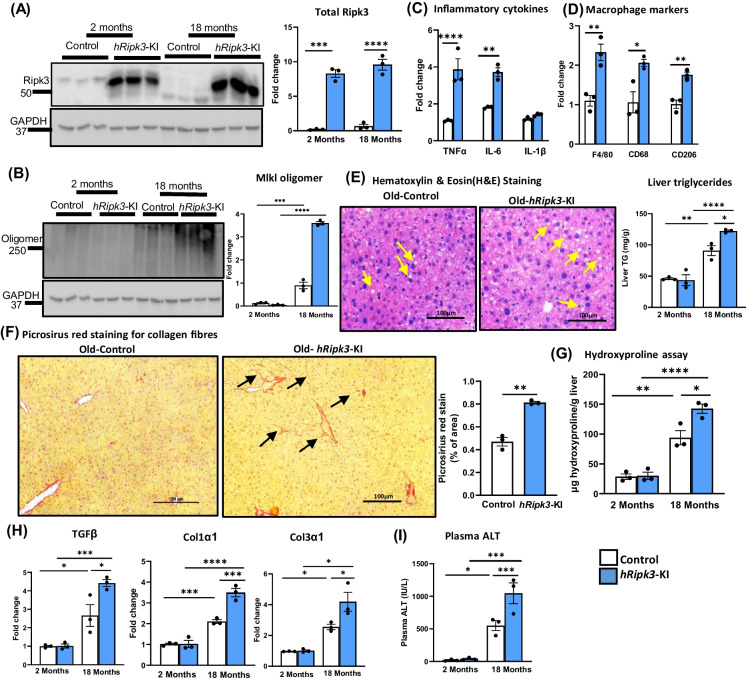
Effect of overexpressing Ripk3 in the livers of old mice. The expression of Ripk3, markers of necroptosis, inflammation, and chronic liver disease were measured in 2- and 18-month-old control (*Ripk3*-KI mice, white bars) or *hRipk3*-KI mice (blue bars). Panel** A**: Western blots of total Ripk3 levels (endogenous and transgene) with the graph to the right showing the quantification of total Ripk3 normalized to β-actin expressed as fold change. Panel** B**: Western blots of MLKL-oligomers with the graph to the right showing the quantification of MLKL-oligomers normalized to GAPDH expressed as fold change. Panel** C**: Transcript levels of TNFα, IL-1β, and IL-6 in 18-month-old mice normalized to β-microglobulin expressed as fold change. Panel** D**: Markers of total macrophages (F4/80), proinflammatory M1 macrophages (CD68), and anti-inflammatory M2 macrophages (CD206) in 18-month-old mice normalized to β-microglobulin expressed as fold change. Panel** E**: Images of H&E staining of sections of liver tissue from 18-month-old mice (scale bar 100 µm) with arrows showing lipid droplets. The graph to the right shows the lipid content in liver expressed as mg of triglycerides/g of liver. Panel** F**: Images of immunohistochemistry staining of the sections of liver tissue from 18-month-old mice (scale bar 100 µm) showing picrosirius red staining with arrows showing chicken wire configuration. The graph to the right shows the quantification of fibrotic areas in the liver. Panel** G**: Hydroxyproline levels expressed as µg of hydroxyproline/g of liver tissue. Panel** H**: Transcript levels of TGFβ, Col1α1, and Col3α1 normalized to β-microglobulin expressed as fold change. Panel** I**: Plasma ALT levels (IU/L). Data were obtained from 3 mice per group, expressed as the mean ± SEM, and statistically analyzed using ANOVA. ****p ≤ 0.0001, ***p ≤ 0.0005, **p ≤ 0.005, *p ≤ 0.05

Aging is associated with increased TNFα [20], oxidative stress [22], and mTOR activation [31], all of which have been shown to induce necroptosis. Therefore, we studied the impact of the lifelong, overexpression of Ripk3 or Mlkl in the livers of a limited number of 18-month-old *hRipk3*-KI and *hMlkl*-KI mice. Because the transgenes are under the control of the Rosa26 promoter, we were unsure what impact age might have on the expression of the transgenes. The data in Fig. 5A show that the overexpression (~ tenfold) of Ripk3 was similar in both young and old *hRipk3*-KI. The western blots and graph in Fig. 5B show that an increase in Mlkl-oligomers was observed in old *Ripk3*-KI mice, which we have previously reported [29]. However, Mlkl-oligomers were dramatically increased (~ fourfold) in old *hRipk3*-KI mice compared to old control, *Ripk3*-KI mice. We observed a significant increase in the levels of TNFα and IL-1β mRNA (Fig. 5C) in the livers of old *hRipk3*-KI mice as well as markers for total macrophages (F4/80), proinflammatory M1 macrophages (CD68), and anti-inflammatory M2 macrophages (CD206) (Fig. 5D). We observed histopathological evidence of liver steatosis (lipid droplets) in the livers of old *hRipk3*-KI mice (Fig. 5E), and the graph shows that liver triglyceride levels that increase with age were significantly higher in the old *hRipk3*-KI mice compared to old, control *Ripk3*-KI mice. Next, we determined the effect overexpressing Ripk3 on the age-related increase in liver fibrosis, which has been observed in mice [22]. Histologically, the old *hRipk3*-KI mice showed the appearance of perisinusoidal/pericellular (chicken wire) fibrosis on picrosirius red staining (Fig. 5F). We also measured several other markers of fibrosis in the liver tissue, e.g., hydroxyproline levels and transcript levels of transforming growth factor β (TGFβ), collagen 1α1 (Col1α1), and collagen 3α1 (Col3α1). Hydroxyproline levels (Fig. 5G) and TGFβ, Col1α1, and Col3α1 expressions (Fig. 5H) all increased with age, which has been observed previously [20]. Importantly, all of these markers were significantly higher in the old *hRipk3*-KI mice compared to control mice. However, we observed no evidence of liver tumors in any of the old mice by gross pathology or histopathology analysis. We also measured plasma ALT levels in the livers of young and old control and *hRipk3*-KI mice. The data in Fig. 5I show that the age-related increase in plasma ALT levels was greater in the *hRipk3*-KI mice.

**Fig. 6:**
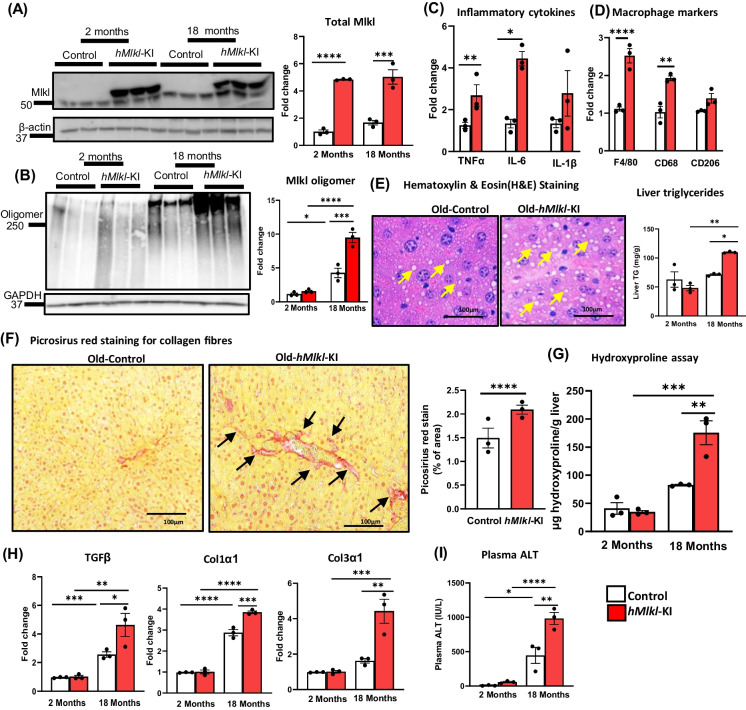
Effect of overexpressing Mlkl in the livers of old mice. The expression of Mlkl, markers of necroptosis, inflammation, and chronic liver disease were measured in 2- and 18-month-old control (*Mlkl*-KI mice, white bars) or *hMlkl*-KI mice (red bars). Panel** A**: Western blots of total Mlkl levels (endogenous and transgene) with the graph to the right showing the quantification of total Mlkl normalized to β-actin expressed as fold change. Panel** B**: Western blots of MLKL-oligomers with the graph to the right showing the quantification of MLKL-oligomers normalized to β-actin expressed as fold change. Panel** C**: Transcript levels of TNFα, IL-1β, and IL-6 in 18-month-old mice normalized to β-microglobulin expressed as fold change. Panel** D**: Markers of total macrophages (F4/80), proinflammatory M1 macrophages (CD68), and anti-inflammatory M2 macrophages (CD206) in 18-month-old mice normalized to β-microglobulin expressed as fold change. Panel** E**: Images of H&E staining of sections of liver tissue from 18-month-old mice (scale bar 100 µm) with arrows showing lipid droplets. The graph to the right shows the lipid content in liver expressed mg of triglycerides/g of liver. Panel** F**: Images of immunohistochemistry staining in the sections of liver tissue from 18-month-old mice (scale bar 100 µm) showing picrosirius red staining with arrows showing chicken wire configuration. The graph to the right shows the quantification of fibrotic areas in the liver. Panel** G**: Hydroxyproline levels expressed as micrograms of hydroxyproline per gram of liver tissue. Panel** H**: Transcript levels of TGFβ, Col1α1, and Col3α1 normalized to β-microglobulin expressed as fold change. Panel** I**: Plasma ALT levels (IU/L). Data were obtained from 3 mice per group, expressed as the mean ± SEM, and statistically analyzed using ANOVA. ****p ≤ 0.0001, ***p ≤ 0.0005, **p ≤ 0.005, *p ≤ 0.05

We next determined whether old *hMlkl*-KI mice also exhibited increased necroptosis. As shown in Fig. 6A, the overexpression of Mlkl was similar in young and old *hMlkl*-KI mice. Mlkl-oligomerization increased with age and was over twofold higher in old *hMlkl*-KI compared to old control, *Mlkl*-KI mice (Fig. 6B). In the old *hRipk3*-KI mice, we observed a significant increase only in TNFα mRNA levels (Fig. 6C); however, markers for total macrophages (F4/80) and proinflammatory M1 macrophages (CD68) were significantly increased in the old *hRipk3*-KI mice compared to old control, *Mlkl*-KI mice (Fig. 6D). We observed histopathological evidence of liver steatosis and increased liver triglycerides in the in the old *hMlkl*-KI mice compared to old control mice (Fig. 6E). Fibrosis was also increased in the livers of the old *hMlkl*-KI mice compared to old mice as shown by the increased: picrosirius red staining (Fig. 6F), hydroxyproline levels (Fig. 6G), and transcript levels for TGF β, Col1α1, and Col3α1 (Fig. 6H). Again, we observed no evidence of liver tumors in any of the old mice by gross pathology or histopathology analysis. The data in Fig. 6I show that plasma ALT levels show a greater age-related increase in the old *hMlkl*-KI mice compared to old control, *Mlkl*-KI mice.

**Fig. 7:**
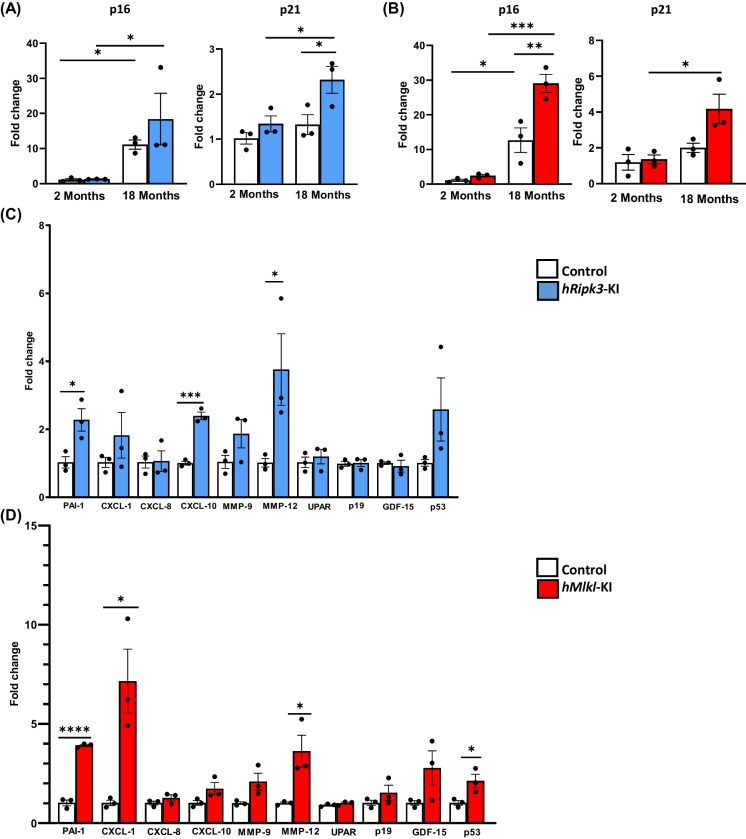
Effect of overexpressing Ripk3 or Mlkl on cell senescence in the livers of old mice. Panel** A**: Transcript levels of p16 and p21 in 2- and 18-month-old control (*Ripk3*-KI mice, white bars) or *hRipk3*-KI mice (blue bars) expressed as fold change. Panel** B**: Transcript levels of p16 and p21 in 2- and 18-month-old control (*Mlkl*-KI mice, white bars) or *hMlkl*-KI mice (red bars) normalized to β-microglobulin expressed as fold change. Panel** C**: Transcript levels of SASP-factors in 18-month-old control (*Ripk3*-KI mice in white bars) or *hRipk3*-KI mice (blue bars) and normalized to β-microglobulin expressed as fold change. Panel** D**: Transcript levels of SASP-factors in 18-month-old control (*Mlkl*-KI mice in white bars) or *hMlkl*-KI mice (red bars) normalized to β-microglobulin expressed as fold change. Data were obtained from 3 mice per group, expressed as the mean ± SEM, and statistically analyzed using ANOVA. ***p ≤ 0.0005, **p ≤ 0.005, *p ≤ 0.05

Because our previous studies showed that blocking necroptosis by Nec-1 s treatment reduced markers of cell senescence in the livers of old mice [20] and *Sod1*^*−/−*^ mice [29], we determined if inducing necroptosis in the livers of old mice accentuated the age-related increase in cell senescence in the liver. The data in Fig. 7A, B show that p16 transcript levels increased with age in control KI and *hRipk3*-KI and *hMlkl*-KI mice. Transcript levels of p21 increased significantly with age only in the *hRipk3*-KI and *hMlkl*-KI mice. Importantly, transcripts for p21 were significantly increased in old *hRipk3*-KI mice compared to control mice. Both p16 and p21 transcripts were increased in old *hMlkl*-KI mice compared old control mice; however, the p21 levels did not reach significance (*p* = 0.06). We also measured the transcripts for markers of the senescent-associated secretory phenotype (SASP). The data in Fig. 7C, D show that the transcripts of several SASP-factors were increased in the livers of the old *hRipk3*-KI and *hMlkl*-KI mice compared to their KI controls. For example, PAI-1, CXCL-10, and MMP-12 were significantly increased in the old *hRipk3*-KI mice. PAI-1, Cxcl-1, and MMP-12 were significantly increased in the old *hMlkl*-KI mice. Transcripts for CxLC-10 and MMP-9 were also increased in the livers of old *hMlkl*-KI mice; however, these increases did not quite reach significance (*p* values ranged from 0.07 to 0.08). Transcript levels for p53 were increased in the old *hRipk3*-KI and *hMlkl*-KI mice compared to control mice; however, this difference was only significant for the old *hMlkl*-KI mice because of the variation in p53 transcript levels in the old *hRipk3*-KI mice. Thus, our data show that specifically inducing necroptosis in the livers of old mice resulted in an increase in various markers of cell senescence.

## Discussion

Research from our group suggests that necroptosis plays an important role in inflammaging. For example, (*i*) necroptosis increases with age and is associated with increased inflammation [20–22]; (*ii*) it is reduced by dietary restriction [19] and increased in *Sod1*^*−/−*^ mice, a mouse model of accelerated aging [22]; and (*iii*) most importantly, inflammation is reduced when necroptosis in inhibited in old [20] or *Sod1*^*−/−*^ mice [22]. The goal of this study was to generate knockin mouse models that would allow investigators to test the effect of inducing necroptosis in a specific cell/tissue on inflammation and aging. Previous studies reported that overexpressing genes involved in necroptosis (e.g., *Ripk3* or *Mlkl*) induced necroptosis and cell death in various types of cells in culture [23–25]. Using a similar strategy, we generated the first knockin mouse models (*Ripk3*-KI or *Mlkl*-KI) that overexpress either Ripk3 or Mlkl under the endogenous Rosa26 promoter when the stop cassette is removed from the transgene after crossing the mice to a Cre transgenic mouse. We chose to develop both *Ripk3*-KI and *Mlkl*-KI models for two reasons. First, these two genes code for proteins that are involved in different steps in necroptosis. Mlkl is the protein that is directly responsible for disrupting the membrane when phosphorylated. Ripk3, when phosphorylated by Ripk1, catalyzes the phosphorylation of Mlkl, which leads to Mlkl forming oligomers. Therefore, it is possible that the overexpression of these two genes might have different abilities to induce necroptosis in vivo. Second, it is important to use both models to rigorously establish that changes observed are due to necroptosis because both Ripk3 and Mlkl affect other pathways. For example, Ripk3 is also involved in apoptosis, NLRP3 activation, and lipid metabolism [32–34], and phosphorylation of Mlkl inhibits autophagy [26]. Showing that the overexpression of both Ripk3 and Mlkl has the same impact on a process is strong evidence that increased necroptosis is responsible for the changes observed in that process.

When the *Ripk3*-KI or *Mlkl*-KI mice were crossed to albumin-Cre transgenic mice, the transgene (either Ripk3 or Mlkl) was specifically expressed only in the liver as would be expected based on the albumin promoter, which drives the expression of the Cre in hepatocytes starting at E14 [27]. Importantly, the *Ripk3*-KI or *Mlkl*-KI mice showed no expression of the transgene in the liver or any other tissue, i.e., the transgenes were not leaky. At 2 or 18 months of age, the levels of Ripk3 or Mlkl were approximately 10- or fourfold greater in the livers of *hRipk3*-KI or *hMlkl*-KI, respectively, compared to control, *Ripk3*-KI or *Mlkl*-KI mice. It is not clear why the expression of these two genes differs because they are expressed by the same Rosa26 promoter. However, it appears that this difference occurs at the transcriptional level because the fold increase in Ripk3 mRNA induced in the *hRipk3*-KI mice was ~ twofold higher than the fold increase in Mlkl mRNA induced in the *hMlkl*-KI mice.

Because the Cre transgene is expressed embryonically (E14), we were initially concerned that the overexpression of either Ripk3 or Mlkl early in life might be lethal or have developmental effects. However, ~ 50% of the mice generated by mating either *Ripk3*-KI or *Mlkl*-KI mice to albumin-Cre transgenic mice were either hRipk3-KI or hMlkl-KI mice as expected, and we observed no developmental abnormalities in either the *hRipk*3-KI or *hMlkl*-KI mice. In addition, the body weights and growth of the *hRipk3*-KI or *hMlkl*-KI mice were similar to the control, KI mice. Although the levels of Ripk3 or Mlkl proteins were dramatically increased in the livers of the *hRipk*3-KI and *hMlkl*-KI mice, we were surprised to find no indication of increased necroptosis in the livers of the 2-month-old mice, e.g., no evidence of Mlkl-oligomers and no increase in markers of inflammation, which are normally associated with increased necroptosis. In retrospect, this result is not that surprising because necroptosis is triggered by TNFα or various stresses, which induce the sequential phosphorylation of Ripk1, Ripk3, and Mlkl. It is likely that necroptosis was not triggered in the young, unstressed *hRipk*3-KI and *hMlkl*-KI mice. Therefore, the overexpression of either Ripk3 or Mlkl did not lead to increase Mlkl-oligomerization in the young mice.

To determine whether stress would lead to increased necroptosis in the young *hRipk*3-KI and *hMlkl*-KI mice, we exposed 2-month-old mice to a single dose of CCl_4_. CCl_4_ is known to induce oxidative stress and further damage cellular systems, leading to hepatotoxic damage [30]. Both *hRipk*3-KI and *hMlkl*-KI mice showed a 2- to threefold increase in Mlkl-oligomers compared to control, *Ripk*3-KI or *Mlkl*-KI mice, 24 h after a relatively mild dose of CCl_4_. The increase in necroptosis (Mlkl-oligomers) was associated with increased markers of inflammation in the liver (e.g., TNFα, IL-1β, total macrophages, and proinflammatory M1 macrophages) and liver damage (plasma ALT levels). Because both the *hRipk*3-KI and *hMlkl*-KI mice showed a similar increase in markers of inflammation in response to CCl_4_ treatment, we have strong evidence that the increase in inflammation induced by CCl_4_ treatment in these mice arose from increased necroptosis. In addition, these data indicate that necroptosis is induced similarly by overexpressing either Ripk3 or Mlkl in mice. In other words, there seems to be no advantage of overexpressing one gene over the other in inducing necroptosis in vivo in the liver.

Our laboratory has shown that necroptosis and markers of inflammation increase with age in the liver of mice e.g., necroptosis (Mlkl-ologimers) increased significantly between 12 and 18 months of age in the liver [20]. Therefore, we studied the impact of overexpressing either Ripk3 or Mlkl over the lifespan of mice. At 18 months of age, the age-related increase in necroptosis (Mlkl-oligomers) was 2- to threefold higher in the old *hRipk*3-KI and *hMlkl*-KI mice compared to the old control, *Ripk*3-KI and *Mlkl*-KI mice. This increase in necroptosis was associated with an increase in markers of inflammation in the liver. Because inflammation plays a role in chronic liver disease [35] and chronic liver disease increases with age, we measured the impact of overexpressing either Ripk3 or Mlkl on steatosis and fibrosis in the old mice. We found that the age-related increase steatosis and fibrosis was significantly increased in the old *hRipk*3-KI and *hMlkl*-KI mice. However, we observed no evidence of liver tumors in any of the old mice, which was not unexpected because fibrosis does not automatically lead to hepatocellular carcinoma [36] and because C57BL/6 J do not normally develop hepatocellular carcinoma [37]. We had previously shown that the increase in necroptosis in the livers of old mice [20] or in *Sod1*^*−/−*^ mice [22] was associated with increased cell senescence, suggesting an interaction between these two cell-fate pathways. The *hRipk*3-KI and *hMlkl*-KI mice allowed us for the first time to directly determine if inducing necroptosis has an impact on cell senescence in a tissue. Our data show that markers of cell senescence and SASP are significantly increased in the livers of the old *hRipk*3-KI and *hMlkl*-KI mice. These data are the first direct evidence showing that increased necroptosis in a tissue can lead to increased cell senescence, suggesting an interaction between necroptosis and cell senescence.

In summary, we describe the first knockin mouse models that can be used to induce necroptosis in vivo. The *Ripk*3-KI or *Mlkl*-KI mice can be used to express either Ripk3 or Mlkl in a specific cell type or tissue when crossed to a Cre transgenic mouse. When crossed to an albumin-Cre transgenic mouse, *hRipk3*-KI and *hMlkl*-KI mice were generated that show increased expression of the transgenes specifically in the liver. When stressed with CCl_4_ or by aging, both *hRipk*3-KI and *hMlkl*-KI mice show similar increases in necroptosis and markers of inflammation. The utility of using these mice to study the impact of necroptosis on aging and age-related diseases is demonstrated by the old *hRipk*3-KI and *hMlkl*-KI mice showing a greater age-related increase in chronic liver disease and cell senescence, which are markers of liver aging.

### Supplementary Information

Below is the link to the electronic supplementary material.

Figure S1 Description of *hRipk3*-KI and *hMlkl*-KI mice. Panel **A**: The transgene construct [SA-loxp-3xstop casset (red) flanked by loxp sites (blue), either flag-tagged (green) *Ripk3* or *Mlkl* cDNA; with BGHpA (light green)] was inserted into the mouse *Rosa26* locus between exon 1 and 2 by CRISPR/Cas9-mediated homology-directed repair. The arrows at the bottom show the sequences used to identify mice containing the transgene. The expression of the Ripk3 or Mlkl transgenes are driven by endogenous Rosa26 promoter when the stop cassette is removed after crossing with Cre transgenic mice. Panel **B**: PCR data show screening of F1 pups for *Ripk3*-KI mice. Panel C: PCR data show screening of F1 pups for *Mlkl*-KI mice. Panel D: Body weights of *hRipk3*-KI mice compared to control, *Ripk3*-KI mice (mean ± SEM for 5 mice per group). Panel **E**: Body weights of *hMlkl*-KI mice compared to control, *Mlkl*-KI mice (mean ± SEM for 5 mice per group). (BMP 2025 KB)

Figure S2 H&E staining of the liver from *hRipk3*-KI and *hMlkl*-KI mice. Images of H&E staining (scale bar:100µm) from sections of liver tissue from 2-months-old *Ripk3*-KI and *hRipk3*-KI mice (Panel **A**) or *Mlkl*-KI and *hMlkl*-KI mice (Panel **B**). (BMP 2025 KB)

Supplementary file3 (DOCX 19 KB)

## Data Availability

The data that support the findings of the study are available in the manuscript and supplementary material of this article. Correspondence and requests for information should be addressed to A.R.

## Abbreviations

*Ripk3*: Receptor-interacting protein kinase 1
*Ripk3*: Receptor-interacting protein kinase 3
*Mlkl*: Mixed lineage kinase domain like
*DAMPs*: Damage-associated molecular patterns
*SASP*: Senescence-associated secretory phenotype
*ALT*: Alanine aminotransferase
*H&E*: Hematoxylin and eosin
