# Supplementary figures and images for: Characterization of novel mouse models to study the role of necroptosis in aging and age-related diseases
Source: GeroScience. 2023 Oct 4;45(6):3241–56. doi: 10.1007/s11357-023-00955-7 (PMC10643444; doi:10.1007/s11357-023-00955-7)

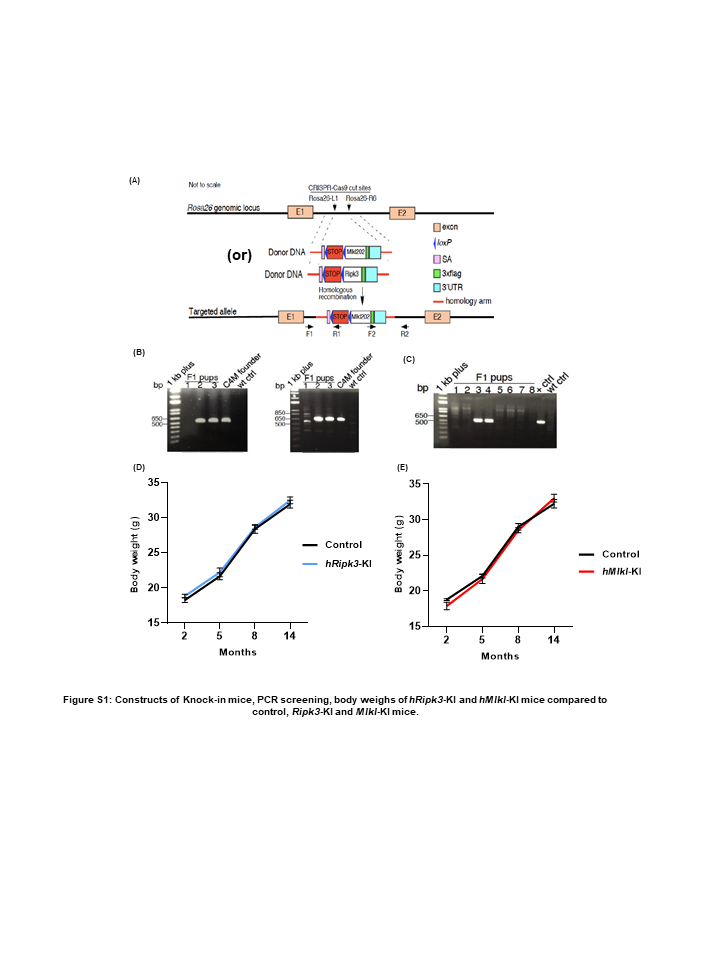

Supplement: Supplementary file 1 — Figure S1 Description of hRipk3-KI and hMlkl-KI mice. Panel A: The transgene construct [SA-loxp-3xstop casset (red) flanked by loxp sites (blue), either flag-tagged (green) Ripk3 or Mlkl cDNA; with BGHpA (light green)] was inserted into the mouse Rosa26 locus between exon 1 and 2 by CRISPR/Cas9-mediated homology-directed repair. The arrows at the bottom show the sequences used to identify mice containing the transgene. The expression of the Ripk3 or Mlkl transgenes are driven by endogenous Rosa26 promoter when the stop cassette is removed after crossing with Cre transgenic mice. Panel B: PCR data show screening of F1 pups for Ripk3-KI mice. Panel C: PCR data show screening of F1 pups for Mlkl-KI mice. Panel D: Body weights of hRipk3-KI mice compared to control, Ripk3-KI mice (mean ± SEM for 5 mice per group). Panel E: Body weights of hMlkl-KI mice compared to control, Mlkl-KI mice (mean ± SEM for 5 mice per group). (BMP 2025 KB) [file 11357_2023_955_MOESM1_ESM.bmp]

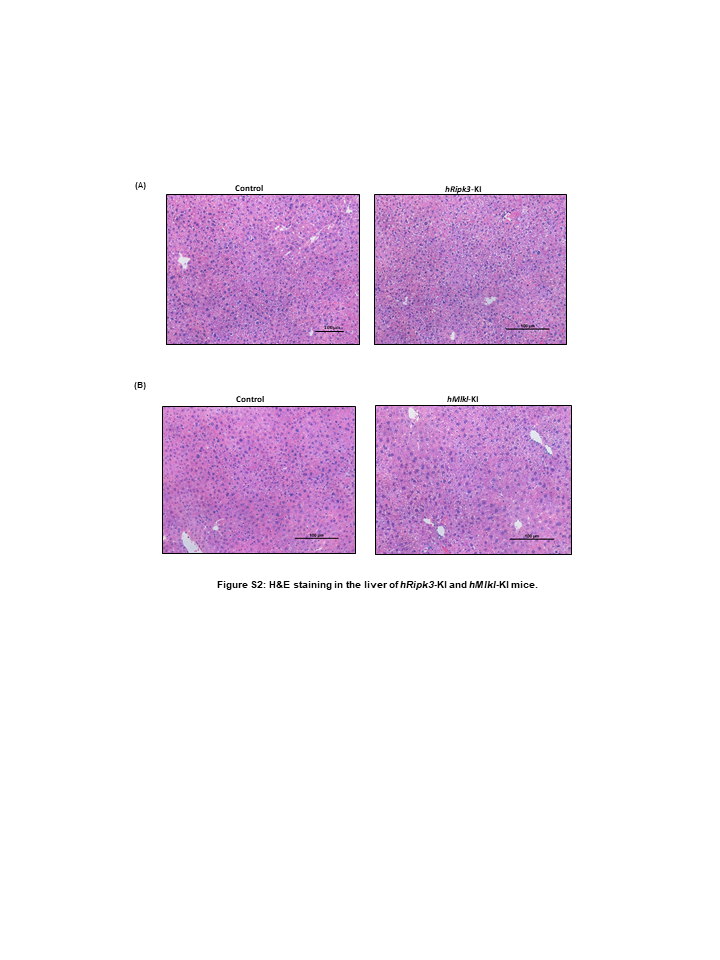

Supplement: Supplementary file 2 — Figure S2 H&E staining of the liver from hRipk3-KI and hMlkl-KI mice. Images of H&E staining (scale bar:100µm) from sections of liver tissue from 2-months-old Ripk3-KI and hRipk3-KI mice (Panel A) or Mlkl-KI and hMlkl-KI mice (Panel B). (BMP 2025 KB) [file 11357_2023_955_MOESM2_ESM.bmp]
