# Supplementary material for: Characterization of novel mouse models to study the role of necroptosis in aging and age-related diseases
Source: GeroScience. 2023 Oct 4;45(6):3241–56. doi: 10.1007/s11357-023-00955-7 (PMC10643444; doi:10.1007/s11357-023-00955-7)
Supplement: Supplementary file 3 — Supplementary file3 (DOCX 19 KB) [file 11357_2023_955_MOESM3_ESM.docx]

**Supplementary Table S1** The sequences of the *Ripk3* and *Mlkl* cDNAs, the loxP site, the stop cassette, and flag-tag in the transgene used to generate the *Ripk3*-KI and *Mlkl*-KI mice.

| **Transgene** | **Sequences** |
| --- | --- |
| *Ripk3* cDNA | ATGTCTTCTGTCAAGTTATGGCCTACTGGTGCGTCAGCGGTTCCTCTGGTGAGCCGTGAAGAACTGAAGAAGCTGGAGTTTGTGGGTAAAGGAGGGTTCGGAGTCGTGTTCCGGGCACACCACAGAACATGGAACCATGATGTAGCAGTCAAGATCGTGAACTCGAAGAAGATATCCTGGGAGGTGAAGGCTATGGTTAATCTTCGTAATGAGAACGTTCTGCTCCTGCTGGGGGTCACTGAGGACCTCCAGTGGGACTTCGTGTCCGGGCAGGCTCTGGTGACAAGATTCATGGAGAATGGCTCCCTCGCAGGGCTGCTGCAACCCGAGTGCCCTCGGCCCTGGCCACTCCTCTGTCGCCTGCTGCAGGAAGTGGTGCTGGGGATGTGCTACCTACACAGCTTGAACCCTCCGCTCCTGCACCGGGACCTCAAGCCCTCTAACATTCTGCTGGATCCAGAGCTCCACGCCAAACTAGCAGATTTTGGCCTGTCCACGTTTCAGGGAGGGTCCCAGTCAGGGTCAGGATCAGGATCAGGATCCAGGGACTCTGGGGGCACCCTAGCGTACTTGGACCCAGAGCTGTTATTTGATGTCAACCTGAAGGCTTCTAAAGCGAGTGATGTCTACAGCTTTGGGATCCTCGTGTGGGCAGTGCTGGCTGGCAGAGAAGCTGAGTTGGTAGACAAGACTTCACTAATCCGGGAAACAGTGTGTGACAGGCAGAGTCGTCCTCCACTGACAGAGCTGCCTCCAGGTAGCCCTGAGACTCCCGGCTTGGAAAAACTGAAGGAGTTAATGATTCATTGCTGGGGTTCCCAGTCCGAAAACAGGCCATCCTTCCAGGACTGCGAACCAAAAACCAATGAAGTTTACAATCTGGTAAAGGACAAGGTAGATGCTGCTGTCTCCGAGGTAAAGCATTATCTGTCTCAGCACAGAAGCAGCGGCAGAAACTTGTCTGCCAGAGAGCCAAGCCAAAGAGGCACAGAAATGGATTGCCCGAGGGAAACCATGGTTTCTAAAATGCTGGACCGCCTGCATTTGGAGGAACCCTCCGGACCAGTTCCTGGAAAATGTCCTGAGAGGCAAGCACAGGACACATCAGTTGGGCCTGCCACACCAGCAAGGACATCTTCTGACCCCGTGGCTGGCACTCCTCAGATTCCACATACTTTACCCTTCAGAGGCACAACACCTGGGCCAGTCTTTACTGAGACTCCCGGTCCTCACCCCCAAAGGAATCAGGGAGATGGAAGACACGGCACTCCTTGGTATCCCTGGACCCCACCGAATCCAATGACAGGGCCACCGGCTCTCGTCTTCAACAACTGTTCTGAAGTGCAGATTGGGAACTACAACTCCTTGGTAGCACCACCAAGAACTACTGCCTCAAGTTCGGCCAAGTATGACCAAGCACAGTTCGGCAGGGGTAGGGGCTGGCAGCCCTTCCACAAGGCTAGCGACTACAAAGACCATGACGGTGATTATAAAGATCATGATATCGATTACAAGGATGACGATGACAAGTAG |
| *Mlkl* cDNA | ATGGATAAATTGGGACAGATCATCAAGTTAGGCCAGCTCATCTATGAACAGTGTGAAAAGATGAAATACTGCCGGAAACAATGCCAGCGTCTAGGAAACCGTGTGCACGGCCTGCTACAGCCTCTCCAGAGACTCCAGGCCCAAGGAAAGAAGAACCTGCCCGATGACATTACTGCTGCCCTGGGCCGTTTTGATGAAGTCCTGAAGGAGGCTAACCAGCAGATAGAAAAGTTCAGCAAGAAGTCCCATATTTGGAAGTTTGTGAGTGTGGGCAATGATAAGATCCTCTTCCATGAAGTGAATGAGAAGCTGAGAGACGTCTGGGAGGAGCTGTTGCTGCTGCTTCAGGTTTATCATTGGAATACCGTTTCAGATGTCAGCCAGCCAGCATCCTGGCAGCAGGAAGATCGACAGGATGCAGAGGAAGACGGAAATGAAAATATGAAAGTTATCCTGATGCAGTTGCAAATTAGCGTGGAAGAAATCAACAAAACCCTGAAGCAATGCTCACTAAAACCCACACAGGAGATCCCACAAGATCTCCAAATCAAGGAGATTCCAAAGGAACATCTTGGACCTCCGTGGACCAAACTGAAGACAAGTAAAATGAGCACCATTTATAGAGGAGAGTATCACAGATCTCCAGTTACCATCAAAGTATTCAACAACCCCCAGGCCGAAAGTGTTGGAATAGTGAGGTTCACTTTCAATGACGAGATCAAAACCATGAAGAAATTCGATTCTCCCAACATCTTGCGTATATTTGGGATTTGCATTGATCAAACAGTGAAGCCCCCTGAGTTCTCCATTGTCATGGAGTACTGTGAACTTGGAACCCTGAGGGAACTGCTGGATAGAGAAAAAGACCTCACAATGAGTGTGCGCAGCCTCCTAGTCCTGAGGGCAGCCAGAGGCTTATACAGGCTACACCATTCGGAAACACTCCACAGAAACATCAGCAGCTCCAGTTTCCTCGTAGCCGGAGGCTACCAAGTAAAGCTTGCAGGATTTGAGTTAAGCAAAACACAGAATTCCATCAGCCGGACAGCAAAGAGCACTAAAGCAGAGAGATCCAGTTCAACGATATATGTCTCCCCTGAGAGACTGAAAAATCCATTTTGCCTTTATGACATAAAAGCTGAAATATATAGCTTTGGAATTGTACTCTGGGAAATTGCCACTGGAAAGATCCCATTTGAAGGCTGTGATTCTAAGAAGATCCGGGAGCTGGTGGCTGAGGACAAGAAGCAGGAACCAGTGGGTCAGGATTGCCCTGAGTTGTTGCGGGAAATCATTAATGAGTGTCGTGCCCATGAGCCCTCCCAACGGCCCTCTGTGGACGGAATCTTGGAGAGACTGTCTGCGGTTGAAGAATCCACGGACAAGAAGGTGGACTACAAAGACCATGACGGTGATTATAAAGATCATGATATCGATTACAAGGATGACGATGACAAGTAA |
| loxP | ATAACTTCGTATAATGTATGCTATACGAAGTTA |
| Stop Cassette (3x) | TCTGTTCCACATACACTTCATTCTCAGTATTGTTTTGCCAAGTTCTAATTCCATCAGAAGCTTGCAGATCTGCGACTCTAGAGGATCTGCGACTCTAGAGGATCATAATCAGCCATACCACATTTGTAGAGGTTTTACTTGCTTTAAAAAACCTCCCACACCTCCCCCTGAACCTGAAACATAAAATGAATGCAATTGTTGTTGTTAACTTGTTTATTGCAGCTTATAATGGTTACAAATAAAGCAATAGCATCACAAATTTCACAAATAAAGCATTTTTTTCACTGCATTCTAGTTGTGGTTTGTCCAAACTCATCAATGTATCTTATCATGTCTGGATCTGCGACTCTAGAGGATCATAATCAGCCATACCACATTTGTAGAGGTTTTACTTGCTTTAAAAAACCTCCCACACCTCCCCCTGAACCTGAAACATAAAATGAATGCAATTGTTGTTGTTAACTTGTTTATTGCAGCTTATAATGGTTACAAATAAAGCAATAGCATCACAAATTTCACAAATAAAGCATTTTTTTCACTGCATTCTAGTTGTGGTTTGTCCAAACTCATCAATGTATCTTATCATGTCTGGATCTGCGACTCTAGAGGATCATAATCAGCCATACCACATTTGTAGAGGTTTTACTTGCTTTAAAAAACCTCCCACACCTCCCCCTGAACCTGAAACATAAAATGAATGCAATTGTTGTTGTTAACTTGTTTATTGCAGCTTATAATGGTTACAAATAAAGCAATAGCATCACAAATTTCACAAATAAAGCATTTTTTTCACTGCATTCTAGTTGTGGTTTGTCCAAACTCATCAATGTATCTTATCATGTCTGGATCCC CATCAAGCTGATCCGGAACCCTTAAT |
| Flag-tag | GACTACAAAGACCATGACGGTGATTATAAAGATCATGATATCGATTACAAGGATGACGATGACAAG |

**Supplementary Table S2** List of primers used to genotype the *Ripk3*-KI and *Mlkl*-KI mice.

| **Sequence** | **Primers** | **Location** |
| --- | --- | --- |
| Rosa/F1a | 5’-TCAGTTGGGCTGTTTTGGAGGCAG-3’ | Outside LHR |
| Stop/R1a | 5’- TCGCAGATCTGCAAGCTTCTGATGG-3’ | Within transgene |
| 3Flag/F2a | 5’-GACTACAAAGACCATGACGGTGATT-3’ | Within transgene |
| Rosa/R2a | 5’-GCCTCGATTTGTGGTGTATGTAAC-3’ | Outside RHR |

**Supplementary Table S3** List of PCR primers used to measure the transcript levels of various genes.

| **Transcript** | **Forward Sequence** | **Reverse Sequence** |
| --- | --- | --- |
| Ripk3 | 5′-GAAGACACGGCACTCCTTGGTA-3′ | 5′-CTTGAGGCAGTAGTTCTTGGTGG-3′ |
| MLKL | 5′-CTGAGGGAACTGCTGGATAGAG-3′ | 5′-CGAGGAAACTGGAGCTGCTGAT-3′ |
| TNFα | 5’-CACAGAAAGCATGATCCGCGACGT-3’ | 5’- CGGCAGAGAGGAGGTTGACTTTCT-3’ |
| IL-6 | 5’-TGGTACTCCAGAAGACCAGAGG-3’ | 5’-AACGATGATGCACTTGCAGA-3’ |
| IL-1β | 5’-AGGGAGTCAACTCATTGGCG-3’ | 5’-TGGCAGAACTGTAGTCTTCGT-3’ |
| F4/80 | 5'-CCCCAGTGTCCTTACAGAGTG-3' | 5'-GTGCCCAGAGTGGATGTCT-3' |
| CD68 | 5’-CCACAGGCAGCACAGTGGAC-3’ | 5’-TCCACAGCAGAAGCTTTGGCCC-3’ |
| CD206 | 5’-ACTACACACTCATCCATTACAACCAA-3’ | 5'-GGCACCTATCACAATCAGGAGGA-3' |
| TGFβ | 5’-ACCATGCCAACTTCTGTCTGGGAC-3’ | 5’-ACAACTGCTCCACCTTGGGCTTG-3’ |
| Col1α1 | 5’-GCTCCTCTTAGGGGCCACT-3’ | 5’-CCACGTCTCACCATTGGGG-3’ |
| Col3α1 | 5’-CTGTAACATGGAAACTGGGGAAA-3’ | 5’- CCATAGCTGAACTGAAAACCACC-3’ |
| p16 | 5'- CCCAACGCCCCGAACT-3’ | 5'- GCAGAAGAGCTGCTACGTGAA-3’ |
| p21 | 5’-GGCAGACCAGCCTGACAGAT-3' | 5’-TTCAGGGTTTTCTCTTGCAGAAG-3' |
| PAI-1 | 5′-GACACCCTCAGCATGTTCATC-3′ | 5′-AGGGTTGCACTAAACATGTCAG-3′ |
| CXCL-1 | 5’-ACCCGCTCGCTTCTCTGT-3’ | 5’-ACCCGCTCGCTTCTCTGT-3’ |
| CXCL-8 | 5’- AGACAGCAGAGCACACAAGC-3’ | 5’- ATGGTTCCTTCCGGTGGT-3’ |
| CXCL-10 | 5’-CCAAGTGCTGCCGTCATTTTC-3’ | 5’-GGCTCGCAGGGATGATTTCAA |
| MMP-9 | 5′-CTGGACAGCCAGACACTAAAG-3′ | 5′-CTCGCGGCAAGTCTTCAGAG-3′ |
| MMP-12 | 5’- TGCACTCTGCTGAAAGGAGTCT-3’ | 5’- GTCATTGGAATTCTGTCCTTTCCA-3’ |
| UPAR | 5′-GCCCAATCCTGGAGCTTGA-3′ | 5′-TCCCCTTGCAGCTGTAACACT-3′ |
| p19 | 5'-GGGTCGCAGGTTCTTGGTC-3' | 5'-AATCTGCACCGTAGTTGAGCA-3' |
| GDF-15 | 5′-GTTAGCCAAAGACTGCCACTG-3′ | 5′-CCTTGAGCCCATTCCACA-3′ |
| p53 | 5'-GGGGAGGAGCCAGGCCATCA-3' | 5'-CTTTCAGTGCAGGGCTTCCTAA-3' |
| HPRT | 5’-CTGGTGAAAAGGACCTCTCG-3’ | 5’-TGAAGTACTCATTATAGTCAAGGGCA-3’ |
| β-microglobulin | 5′-CACTGACCGGCCTGTATGC-3′ | 5′-GGGTGGCGTGAGTATACTTGAAT-3′ |
| β-actin | 5′-ATGGATGACGATATCGCTG-3′ | 5′-GTTGGTAACAATGCCATGTTC-3′ |
